# The Influence of Melatonin Treatment in the Vinification of Feteasca Neagra and Cabernet Sauvignon Wines on the Profile of Polyphenolic Compounds and Antioxidant Activity

**DOI:** 10.3390/antiox12061214

**Published:** 2023-06-04

**Authors:** Sandra A. V. Eremia, Camelia Albu, Gabriel-Lucian Radu, Andreia Alecu, Elena Brinduse

**Affiliations:** 1 Centre of Bioanalysis, National Institute of Research and Development for Biological Sciences–Bucharest, 296 Splaiul Independentei, 060031 Bucharest, Romania; 2 Institute for Research and Development for Viticulture and Wine Making, 2 Valea Mantei, Valea Calugareasca, 107620 Prahova, Romania

**Keywords:** melatonin treatment, winemaking process, synergistic effects, polyphenolic compounds, phenylpropanoid metabolism, antioxidant activity, Feteasca Neagra, Cabernet Sauvignon

## Abstract

Until recently, the main antioxidant role among wine constituents was attributed to polyphenolic compounds, but once the presence of melatonin in wines was confirmed, an interesting new field of research opened up due to its possible synergistic effects with other antioxidants in the winemaking process, which may lead to a change in the profile of polyphenolic compounds and antioxidant activity. In order to investigate the evolution of active principles from the phenylpropanoid metabolism associated with the synergistic effects of melatonin, for the first time, a melatonin treatment was performed in the pre-stage of the different winemaking processes of Feteasca Neagra and Cabernet Sauvignon wines with different melatonin concentrations. After comparing the acquired results for the evolution of the polyphenolic compound profile and antioxidant activity of treated wines, we ascertained an increase in the antioxidant compound concentrations, especially in resveratrol, quercetin, and cyanidin-3-glucoside, directly proportional to the used melatonin concentration; an intensification in activity of PAL and C4H enzymes; and the modification in the expression of specific anthocyanin biosynthesis genes, especially UDP-D-glucose-flavonoid-3-*O*-glycosyltransferase. It was also shown that the application of melatonin in the pre-stage of the winemaking process can be successfully used to obtain red wines with increased antioxidant activity (almost 14%).

## 1. Introduction

**Figure 1:**
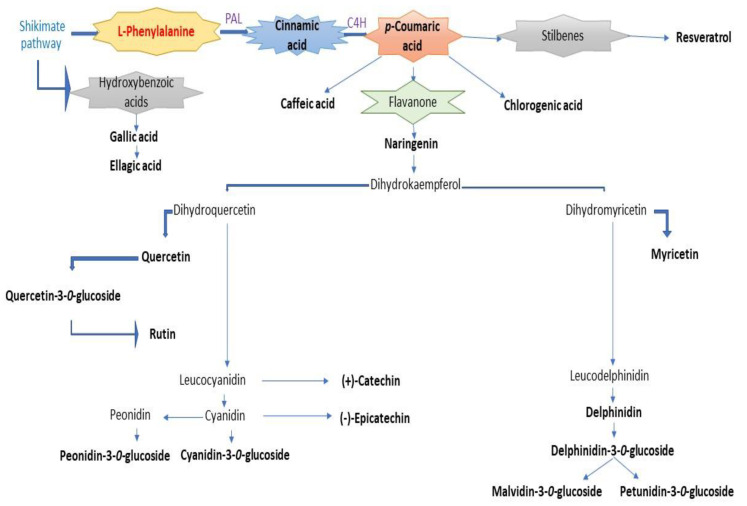
Biosynthesis pathways of various polyphenolic compounds in red wines involving the shikimate/phenylpropanoid metabolism.

Red wines are alcoholic beverages that contain a variety of natural bioactive compounds with antioxidant properties. The most important antioxidant role among wine components is attributed to polyphenols, but they are also responsible for the organoleptic properties of red wine: the colour [1], bitterness, astringency [2], taste [3], and aroma [4]. These compounds with different chemical functions and structures are secondary metabolites in red wines, biosynthesised via the shikimate pathway and/or phenylpropanoid metabolism [5]. The major steps in phenylpropanoid metabolism are the reactions in which phenylalanine undergoes deamination catalysed by phenylalanine ammonia lyase (PAL) to yield trans-cinnamic acid and the hydroxylation of cinnamic acid catalysed by cinnamic acid 4-hydroxylase (C4H) to yield p-coumaric acid [6]. The p-coumaric acid is an essential compound in further reactions involved in the biosynthesis of various classes of polyphenolic compounds [7] found in red wines (Figure 1). Red wines contain different types of polyphenols that are extracted from the grapes during winemaking. For example, the phenolic acids (hydroxybenzoic acids and hydroxycinnamic acids) are present in the pulp, seeds, and skins of the grapes [8], with the skins [9], the stilbenes [10], and flavonols [11] found in the skins, the flavan-3-ols concentrated in all the solid parts of the grapes (skins, stems and seeds) [12], and the anthocyanins mainly enriched in the skins [13]. The grape varieties, the yeast used in the fermentation of the wine, the contact with the wood, and the ageing processes all have an influence on the concentration and composition of the polyphenolic compounds in the finished wine [14]. Thus, during the winemaking process, the biosynthesis and accumulation of secondary metabolites due to phenylpropanoid pathways can be observed, leading to a change in the composition of polyphenolic compounds.

Melatonin, an important indoleamine hormone with potent antioxidant activity [15], can directly scavenge free radicals and stimulate the activity of antioxidant enzymes, suppress prooxidant enzymes, and reduce the formation of radicals by improving mitochondrial function [16]. The presence of melatonin in wines is due to the fact that it is present in grapes [17] and that it is biosynthesised by yeasts from its precursors tryptophan and serotonin at some stages of the winemaking process, mainly during alcoholic fermentation [18]. Until recently, the monitored components with antioxidant activity in wines were polyphenols, but once the presence of melatonin in wines was confirmed [19], attention was also focused on this antioxidant compound due to its possible synergistic and additive effects with other antioxidants in the winemaking process. The increase in antioxidant activity induced by melatonin is not only related to the endogenous antioxidant capacity of melatonin but also to the enhancement of polyphenolic compounds and to their complicated reactions [20]. Melatonin may have a synergistic effect with other antioxidants naturally present in wines, such as resveratrol [21], resulting in a higher cytoprotective effect against oxidative stress [22]. Melatonin in tea leaves has been reported to enhance the production of catechins, as their biosynthesis depends on carbon flux to the secondary metabolic pathway and melatonin enhances this flux by accelerating photosynthesis [23]. In addition, melatonin treatment has been observed to upregulate the expression of genes involved in anthocyanin biosynthesis, increasing total anthocyanin production in cabbage [24] and pigment accumulation in tomato [25]. Exogenous melatonin application to grape berries in the pre-season period plays an important role in increasing the activities of enzymes, including PAL [26], and in inducing the expression of genes related to anthocyanin biosynthesis [27], leading to the biosynthesis of rutin, (+)-catechin, (−)-epicatechin [28], syringic acid, and coumaric acid [20], as well as the accumulation of anthocyanins and other related substances in grape berries. However, the role of melatonin in phenylpropanoid metabolism is unclear. Studies are still in the early stages, and it is not clear how melatonin treatment can change the content and/or composition of phenolic compounds and the antioxidant activity of red wines. In present, melatonin is not on the list of International Organisation of Vine and Wine (OIV) of admitted compounds and their status as additives or processing aids and use levels or residual limits [29] but has been approved as an ingredient of foodstuffs in Europe, Canada, and the USA [30]. To date, there are no studies using melatonin treatment in winemaking and following the evolution of the polyphenolic profile and antioxidant activity during the ageing process of the wines to understand how melatonin might regulate polyphenol metabolism.

The aim of this study was to investigate the influence of melatonin treatment during winemaking on the profile of polyphenolic compounds and antioxidant activity in Feteasca Neagra, and Cabernet Sauvignon red wines. In order to understand how melatonin might regulate the polyphenol metabolism, the appropriate spectrophotometry and high-performance liquid chromatography (HPLC) methods were used to monitor, after 3 months of ageing, the evolution of the active compounds related to the synergistic effects of melatonin treatment both in the improved red wines and in the control wines.

## 2. Materials and Methods

### 2.1. Reagents

Polyphenol compounds, caffeic acid (C0625), chlorogenic acid (C3878), p-coumaric acid (C9008), (−)-catechin (C0567), (+)-catechin (43412), naringenin (N5893), quercetin (Q4951), quercitrin (Q3001), quercetin 3-β-D-glucoside (17793), resveratrol (R5010), rutin (78095), 2,2-azinobis(3-ethylbenzothiaziline-6-sulfonate) (ABTS), 6-hidroxi-2,5,7,8-tetrametillchroman-2-carboxilic acid (Trolox), 2,2-difenil-1-picrilhidrazil (DPPH), sodium carbonate (Na_2_CO_3_), potassium persulfate (K_2_S_2_O_8_), and Folin and Ciocalteu’s phenol reagent (FCR) were obtained from Sigma-Aldrich. Gallic acid (48630), trans-cinnamic acid (96340), ellagic acid (45140), (−)-epicatechin (68097), myricetin (70050), and DL-phenylalanin (78040) were purchased from Fluka, From Roth, we used delphinidin (4537.1), malvidin (6140.1), and peonidin-3-glucoside (1619.1), and from PhytoLab, we used petunidin-3-glucoside (89755). Anthocyanins, cyanidin-3-glucoside, delphinidin-3-glucoside, and malvidin-3-glucoside were purchased from Polyphenols AS, Sandnes, Norway. Taking into account the chemical composition of the wine, the major components were water (86%) and ethanol, but as the specific solubility of each polyphenolic compound and some compounds are poorly soluble in water, the stock solutions, 1 mg mL^−1^, were prepared in ethanol (Riedel-de Haen, Berlin, Germany) and then diluted in water, protected from light, and stored at 4 °C. All other used reagents, MeCN (Riedel-de Haen) and methanol (Riedel-de Haen), were of chromatographic or analytical purity. The ultra-pure water was obtained with an Elix 3 (Millipore, Darmstadt, Germany) system. 

### 2.2. Wine Sampling

Two different wine samples, Feteasca Neagra (FN) and Cabernet Sauvignon (CS) from Valea Calugareasca, Romania, were evaluated. FN and CS grapes were randomly harvested at technological maturity when the content of soluble sugars reached 210.1 ± 0.05 g L^−1^ for FN and 212.4 ± 0.14 g L^−1^ for CS. Total acidity had the value of 4.48 ± 0.12 g L^−1^ for FN and 4.10 ± 0.06 g L^−1^ for CS. The grapes were sulfited (50 mg L^−1^ SO_2_), destemmed, and crushed. A total of 20 L experimental variant from the obtained must was transferred to the glass fermentation vessels of 25 L volume. Commercial *Saccharomyces cerevisiae* Actiflore F 33 (20 g hL^−1^) was applied in the must to start the fermentation. Two replicates were conducted for each variant. The wines were obtained by the traditional process of maceration, in which FN was obtained by racking and CS by pumping over. Melatonin was applied at the pre-winemaking stage, with 50 µg of melatonin added to 1 kg of must from CS and 500 µg of melatonin added to 1 kg of must from FN. Prior to HPLC analysis, samples were filtered into HPLC vials with a 25 mm, 0.2 µm Syringe-Driven Philtre Unit (PVDF, Agilent, Santa Clara, CA, USA) and injected into the HPLC system.

### 2.3. Trolox Equivalent Antioxidant Capacity (TEAC) Assay Using ABTS·^+^

The Trolox equivalent test for antioxidant capacity requires the use of ABTS·^+^, a radical cation produced by oxidation of ABTS (7 × 10^−3^ M) in water with K_2_S_2_O_8_ (2.5 × 10^−3^ M). The ABTS·^+^ is a long-lived radical that stabilises after at least 20 h at room temperature in the dark. The working solution is prepared by diluting the stock solution to 1:40 *v*/*v* and then mixing with the sample and water prior to the measurements at a ratio of 25:1:4, ABTS·^+^/sample/solvent, *v*/*v*/*v*. The protocol is based on absorbance measurement at 735 nm using a Thermo Evolution 260 Bio Spectrophotometer (Thermo Fischer Scientific, Waltham, MA, USA). In the presence of an antioxidant compound, the measured absorbance for the radical cation decreases in direct proportion to the antiradical capacity. The results were expressed in relation to Trolox equivalent (8.5 × 10^−6^ M) [31], a compound that is considered a reference antioxidant.

### 2.4. TEAC Assay Using DPPH^•^

The protocol requires the use of DPPH^•^ (2.5 × 10^−4^ M), a stable radical obtained by dissolving DPPH in a suitable solvent (ethanol). The stock solution is mixed with the sample in a volumetric ratio of 1:0.1:1.9, DPPH^•^/sample/solvent, *v*/*v*/*v*. The protocol is based on the measurement of the decrease in DPPH absorption maximum at 515 nm using a Thermo Evolution 260 Bio Spectrophotometer (Thermo Fischer Scientific, Waltham, MA, USA). In the presence of an antioxidant compound, the absorption of DPPH- decreases in direct proportion to the antiradical capacity. The results were expressed as Trolox (8.5 × 10^−6^ M) equivalent [32].

### 2.5. Folin–Ciocalteu Assay

The phenol reagent of Folin–Ciocalteu was diluted 10-fold in distilled water and mixed with the phenol compound 5:1, *v*/*v*. After 10 min, Na_2_CO_3_ (7.8%) was added to obtain a blue complex between the Folin–Ciocalteu reagent and the phenols in the alkaline medium [33]. After 1 h, the absorbance value of the blue complex was measured with a Thermo Evolution 260 Bio spectrophotometer (Thermo Fischer Scientific, Waltham, MA, USA) at 766 nm, which was proportional to the concentration of the phenolic compounds present in the sample. The values obtained are expressed in relation to gallic acid, which is considered the reference polyphenol. Therefore, it was necessary to establish the calibration curve for gallic acid (linear range: 0.01–0.1 mg mL^−1^).

### 2.6. HPLC Analysis

HPLC analysis was achieved using an HPLC Shimadzu composed of a SIL-20AC autosampler, a DGU-20A degasser, two LC-20AD pumps, a CTO-20A column oven, and LC Solution software var 5.1. The HPLC was coupled to a mass spectrometer detector, LCMS-2010, with an ESI interface and the following parameters: nebulization gas (N_2_) flow rate, 1.5 L min^−1^; CDL temperature, 200 °C; heat block temperature, 200 °C; detector voltage, 1.8 kV; interface temperature, 250 °C; and interface voltage, 4 kV.

The identification and quantification of phenolic acids, flavanols, flavonols, and stilbenes was performed on a Kromasil 100-3.5-C18 2.1 × 100 mm column and with an elution gradient of mobile phase (formic acid in water, pH = 3, solvent A and formic acid in MeCN; pH = 3, solvent B; 0–20 min for 5–30% solvent B, 20.01–40 min for 30% solvent B, 40.01–50 min for 50% solvent B, 50.01–52 min for 50–5% solvent B, 52.01–62 min for 5% solvent B) and a gradient of flow rate (0.1 mL min^−1^ from 0–5, 15.01–35, and 60.01–62 min, and 0.2 mL min^−1^ between 5.01–15 and 35.01–60 min) [34]. Due to the specificity of the compounds of interest, the mass spectrometric detection was carried out using negative ionisation mode. For quantitative analysis, we used selected ion monitoring (SIM) mode, and the corresponding peaks of the polyphenol compound fragment ions ([M-H]^−^: 163, 147, 169, 179, 227, 271, 289, 301, 317 353, 447, 463, and 609) were obtained.

The identification and quantification of monomeric anthocyanidins and anthocyanins was carried out with a Kromasil 100-3.5-C18 4.6 × 50 mm column at 40 °C temperature, an elution gradient of mobile phase (5% formic acid in water, solvent A, and 5% formic acid in methanol, solvent B; 0–3 min 0% solvent B, 3–15 min 0–20% solvent B, 15–30 min 20–50% solvent B, 30–36 min 0% solvent B, 36–47 min 0% solvent B) and a 0.2 mL min^−1^ flow rate [35]. The mass spectrometer detector used positive ionisation mode, and the corresponding peaks of the anthocyanidin/anthocyanin fragment ions ([M-H]^+^: 303, 331, 463, 449, 465, 479, and 493) in SIM mode were obtained.

The phenylalanine analysis was accomplished with a Kromasil 100-3.5-C18 2.1 × 100 mm column, an elution gradient of mobile phase (water, solvent A, and methanol, solvent B; 0–5 min 5% solvent B, 5–20 min 0–30% solvent B, 20.01–25 min 5% solvent B) and a gradient of flow rate (0.1 mL min^−1^ from 0 to 5 and 15.01 to 25 min, and 0.2 mL min^−1^ between 5.01 and 15 min). The mass spectrometer detector used positive ionisation mode, and fragment ions with [M-H]^+^: 166 was monitored.

### 2.7. Statistical Analysis

Each concentration of the standards and wine sample was tested in triplicate. The results both for the HPLC analyses and for the evaluation of the antioxidant activity are expressed as the mean ± standard deviation (SD). For the statistical analysis, we used the software Microsoft Office Excel 2019.

## 3. Results and Discussion

### 3.1. The Evolution of the Polyphenolic Compound Profile in FN and CS Wines

Experiments were first conducted to determine the performance characteristics of the HPLC methods required for analytical analysis to evaluate the polyphenol profile. Calibration curves were obtained for seven different concentrations in the range of 0.5–50 µg mL^−1^ or limit of detection (LoD), and the limit of quantification (LoQ) was calculated as 3 and 10 times the signal-to-noise ratio. The values obtained for the correlation coefficients (R), range of response, LoD, and LoQ confirm that these methods are suitable for the analysis of polyphenolic compounds in red wine samples (Table 1 and Table 2).

In order to achieve the aim of this work, a treatment with melatonin in the must at the preliminary stage of the winemaking process was carried out in different quantities and different red wine varieties, namely, 50 µg of melatonin in 1 kg of must CS and 500 µg of melatonin in 1 kg of must FN. In addition, control wines were made under the same conditions, from the same grape varieties and with the same maceration process, but without melatonin, in order to be able to monitor the polyphenolic profile as a function of the grape variety and the winemaking process. Since the aim was to understand how the melatonin treatment applied affects fermentation and development during ageing, the antioxidant bioactive compounds of the wines were analysed qualitatively and quantitatively after 3 months of ageing. The results of the HPLC-MS analyses are shown in Table 3 and Table 4.

Gallic acid, a hydrobenzoic acid, is first synthesised via the shikimic acid pathway in grapes or wine. Its precursor is 3-dehydroquinic acid, which is involved in the third step of the shikimate pathway. Over time, an increase in gallic acid is observed during wine ageing due to the dissociation of certain compounds such as the hydrolysis of galloylated proanthocyanidins or wine copigments [36]. Gallic acid, in combination with simple sugars, leads to hydrolysable tannins or to ellagic acid, or, after the addition of a molecule of phosphoenolpyruvate and other intermediate steps, is converted into phenylalanine, the key compound of the phenylpropanoid metabolism [37]. As shown in Table 3, gallic acid was not the most abundant phenolic compound in the wines for the time being, probably due to the fact that the wines were analysed early in the winemaking process (3 months of ageing) and the hydrolysis of galloylated tannins or copigments requires more time. Furthermore, a decrease in concentrations was observed in the treated wines compared to the control wines, although this decrease was almost insignificant (≈4%) in FN, the wine treated with the highest concentration of melatonin. Possibly, in the presence of melatonin, the transformation of gallic acid into ellagic acid is intensified, as the must represents a favourable environment containing simple sugars, which explains the higher levels of ellagic acid in the treated wines.

From the results obtained in the treated wines in the first steps of phenylpropanoid metabolism, there was an increase in the concentration of *trans*-cinnamic acid by ≈38% in CS and ≈46% in FN, but also of *p*-coumaric acid by ≈25% in CS and ≈68% in FN. These values, together with the results of quantitative phenylalanine analysis, show that the α-amino acid concentration in both melatonin-enriched wines decreased by ≈15%, showing that the presence of melatonin stimulated the activity of the enzymes PAL and C4H, which promoted the accumulation of phenolic compounds in the wines. These results are in agreement with those obtained as a consequence of the application of exogenous melatonin on fruits, in which the increase in the activity of enzymes related to phenylpropane metabolism, PAL and C4H, was reported [38]. The presence of melatonin in wines led to the deamination of phenylalanine to trans-cinnamic acid catalysed by PAL, as well as the hydroxylation of cinnamic acid to p-coumaric acid catalysed by C4H, with a higher chemical reactivity observed in FN wine treated with a greater amount of melatonin than CS wine.

The treatment with melatonin induced the expression of stilbene synthase genes, located in chromosome 16 [39], and enhanced the content of resveratrol in wines. The increase in the concentration of resveratrol was due the intensification of grapevine resveratrol synthase gene activities in the presences of *Saccharomyces cerevisiae* and a necessary level of *p*-coumaric acid precursor in the must. Similarly, melatonin application increased the chalcone synthase involved in naringenin biosynthesis [40]. The chalcone synthase genes are present in the genome of all plants, while stilbene synthase genes have been found in a limited number of plant species. We ascertained the intensification of resveratrol, namely, naringenin biosynthesis from *p*-coumaric acid in the treated wines with 42% in CS and 50% in FN in the case of resveratrol, as well as with 32% in CS and 66% in FN in the case of naringenin, in comparison with the values obtained for the control wines. The transformation of *p*-coumaric acid into caffeic acid and chlorogenic acid requires the application of at least 500 µg of melatonin in 1 kg of must, with these biosyntheses being found only in FN wine.

After treatment with melatonin, there is a functional dependence between the administered concentration of melatonin and the concentration of myricetin or the concentration of quercetin compounds, secondary metabolites in red wines that can be biosynthesised by phenylpropanoid metabolism involving naringenin, the common flavanone precursor for all flavonols and implicitly for dihydrokaempferol. Production levels may have been increased by facilitating hydroxylation steps carried out by enzymes that require a reducing force provided by a suitable redox partner, in this case, melatonin. Starting from dihydrokaempferol, two hydroxylations take place in myricetin, whereas only one takes place in quercetin. The myricetin concentration in the wine FN was 26% significantly higher than the 13% in the wine CS. Furthermore, a significant positive correlation was found between melatonin and the production of the quercetin compounds quercetin and quercetin 3-β-D-glucoside. In FN wine, an increase of ≈36% was found, while in CS wine, where a much lower concentration of melatonin was applied, only a 5% improvement was found. The slight increase in the concentrations of rutin or quercitrin in the treated wines can also be explained by the fact that these compounds are affected by the ageing process of the wine and their concentration decreases significantly after 90 days of ageing [41]. It is known that (+)-catechin and (−)-epicatechin serve as precursors for proanthocyanidins and that melatonin acts as a modulator of proanthocyanidin biosynthesis rather than a trigger [42]. The modulation probably occurs through the regulation of the initial stages, namely, the conversion of catechins, as evidenced by the positive deviations of up to 27% in the concentrations of catechins in the treated wines.

Anthocyanins are synthesised in the phenylpropanoid pathway under the complex control of all structural enzymes and numerous regulatory genes at the transcriptional level. In grapes, the sugar moiety is usually glucose, and the UDP-D-glucose flavonoid 3-*O*-glycosyltransferase can *O*-glycosylate the anthocyanidins via glycosidic bonds at the C3 positions with the addition of glucose molecules to 3-*O*-monoglycosides. Therefore, the most important anthocyanins in wines are the 3-*O*-monoglucosides of cyanidin, peonidin, delphinidin, petunidin, and malvidin, but not of pelargonidin, which is present in certain wine varieties in traces [43].

Leucocyanidins or cyanidin in wines were chemically converted not only to catechins but also to cyanidin-3-glucoside and peonidin-3-glucoside by C3 glucosylation reactions catalysed in particular by the activity of anthocyanidin/anthocyanin glycosyltransferases. Based on the data obtained after HPLC analysis of anthocyanins, it can be concluded that melatonin enhanced the gene expression associated with anthocyanin biosynthesis, particularly in the biosynthesis of cyanidin-3-glucoside. In the case of cyanidin-3-glucoside, an increase of almost 77% in FN wine and 40% in CS wine was observed in the treated wines, and in the case of peonidin-3-glucoside of 24% in FN treated wine and 14% in CS treated wine. Moreover, in this case, the existence of a directly proportional relationship between the melatonin concentration in the wines and the intensification of anthocyanin biosynthesis in these wines was confirmed.

The HPLC results also show that the differences between the delphinidin concentration in the treated wines and the control wines were insignificant, but there were variations between the delphinidin-3-glucosidei concentrations. An increase in the concentration level of 22% in the FN wine and 18% in the CS wine was observed, which proves that the application of melatonin in wines leads to an enhancement of the enzyme activity of UDP-D-glucose flavonoid 3-*O*-glycosyltransferase. Delphinidin 3-*O*-glucoside underwent two successive methylations, mediated by *O*-methyltransferases and with S-adenosylmethionine as a methyl donor, at the oxygen in the 3′- and 5′-positions, giving successively petunidin 3-*O*-glucoside and the malvidin 3-*O*-glucoside. An increase of about 13% in the petunidin 3-glucoside concentration was observed in the two treated wines. For malvidin-3-glucoside, the concentrations were similar in both the control and treated wines. This indicated that the synthesis of anthocyanins in the analysed wines by two successive methylations was not influenced by melatonin. Normally, the most abundant monomeric anthocyanin in young red wines is malvidin-3-*O*-glucoside [44]. In our study, the major monomeric anthocyanin was delphinidin-3-*O*-glucoside, which was due to the early analysis of the wines, i.e., 3 months after the start of winemaking, when the reactions involved in anthocyanin metabolism were still in progress.

The obtained results are in agreement with those previously published [27], and it can be concluded that the exogenous application of melatonin can significantly alter the expression of specific anthocyanin biosynthesis genes, in particular, UDP-D-glucose flavonoid 3-*O*-glycosyltransferase, as well as the production and accumulation of anthocyanins in wines.

### 3.2. The Evolution of Antioxidant Activity in FN and CS Wines

When studying the profile of polyphenolic compounds and the antioxidant activity of wines, it is advisable to use more than one method, as each method provides different information. Certain antioxidants do not react with certain oxidising species, but they do with some others, and different methods provide complementary information. DPPH and ABTS are the most commonly used methods for assessing antioxidant capacity. The differences between the results of these analyses depend on the chemical reagent used. Both assays are based on electron transfer and involve the reduction of an oxidant. When assessing the antioxidant capacity of wines, the correlation between these assays and the total phenolic content (TPC) can be a useful tool. Therefore, to complete the polyphenolic profile obtained from the analyses of HPLC-MS, the DPPH and ABTS assays were used to estimate antioxidant efficiency, and the Folin–Ciocalteu reagent was used for total phenolic content.

The antioxidant capacity of different types of wines was measured on the basis of the study of the effect of a sample containing antioxidants on the oxidative degradation of a test system that should be of physiological significance. In order to evaluate the antioxidant capacity of wines, the technical and operational parameters such as the specific measurement wavelength, the maximum absorbance value, and the dilution factor used according to the sample content were established.

**Figure 2:**
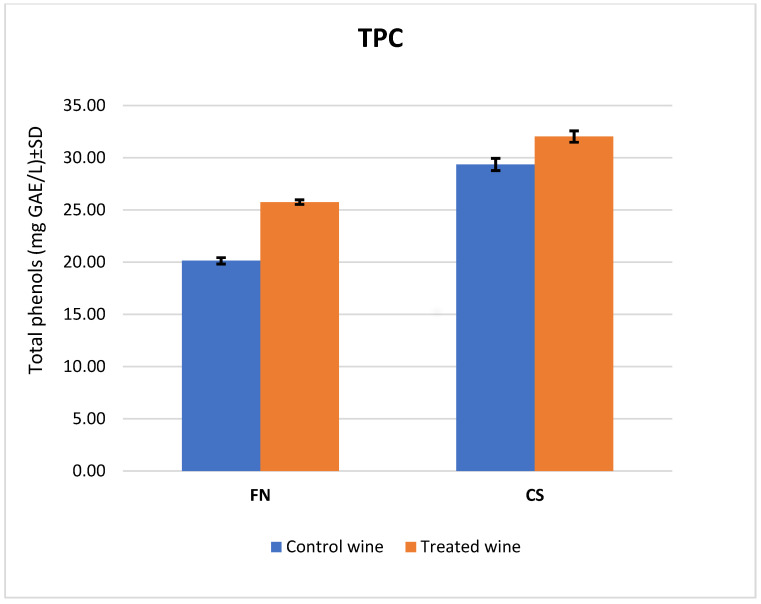
The comparison of total phenolic content in CS and FN wines.

It can be noted that the results obtained for the total TPC (Figure 2) are in agreement with the results of the HPLC analyses of the polyphenolic compounds. As in the HPLC analyses, higher content values were obtained for the wines treated with melatonin, 24% in the case of the FN wine and 9% each in the case of the CS wine. The values for the gallic acid equivalent (GAE) also agreed with those of the HPLC analysis, giving 32.3 ± 0.54 GAE mg L^−1^ for the treated CS wines and 25.75 ± 0.20 GAE mg L^−1^ for the treated FN wines.

**Figure 3:**
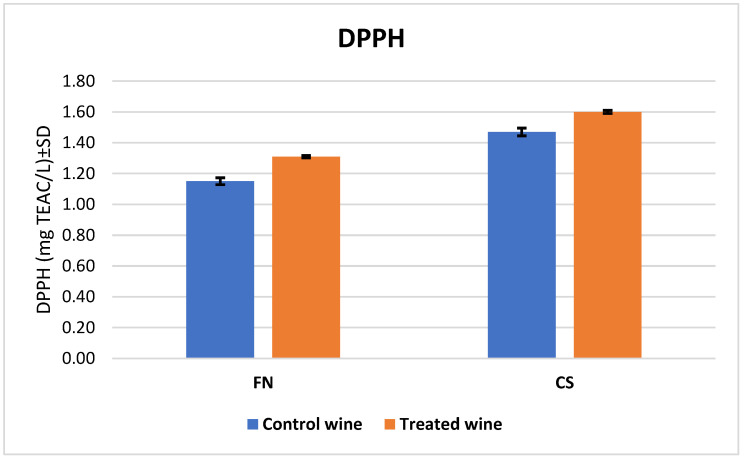
The comparison of results obtained from the DPPH assay for CS and FN wines.

Figure 3 shows that melatonin use increased the ability to scavenge DPPH free radicals, with the highest increase for wine FN at 14% and for wine CS at 9%. Moreover, an agreement with the results for TPC was observed, and the data values found were higher for CS wines (1.47 ± 0.03 TEAC mg L^−1^ for the control wine and 1.60 ± 0.01 TEAC mg L^−1^ for the treated wine) than for the FN wines (1.15 ± 0.02 TEAC mg L^−1^ for the control wine and 1.31 ± 0.01 TEAC mg L^−1^ for the treated wine).

**Figure 4:**
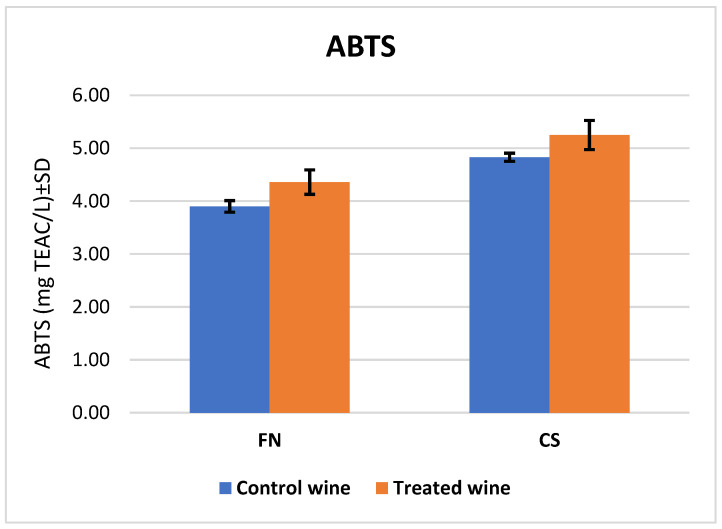
The comparison of results obtained from the ABTS assay for CS and FN wines.

The values for ABTS·+ radical scavenging capacity of the individual wine groups did not differ significantly, for FN at 3.9 ± 0.11 TEAC mg L^−1^ for the control wine and 4.36 ± 0.23 TEAC mg L^−1^ for the treated wine, and for CS at 4.83 ± 0.08 TEAC mg L^−1^ for the control wine and 5.25 ± 0.28 TEAC mg L^−1^ for the treated wine (Figure 4). The differences correlated with the results obtained for TPC. There was an intensification by melatonin treatment of the wines by almost 12% in the FN wine and 9% in the CS wine.

After comparing the results obtained for the evaluation of antioxidant activity in the FN and CS wines, it can be noted that the CS treated wine sample with the highest content of phenolic compounds also had the highest antioxidant activity, proving that TPC and antioxidant activity measured by both the DPPH and ABTS assays were significantly correlated. This correlation confirms the fact that the antioxidant activity in these wines is mainly due to the phenolic compounds. The expected contribution of the antioxidant activity of melatonin cannot be significant if we take into account that the melatonin concentration used in the wines was in the range of µg L^−1^; the pH in the wines was below the physiological level, which drastically changed the ABTS**^.^**^+^ scavenging ability of melatonin; and the short time to record absorption for the radical cation decreased, with the interaction of melatonin with ABTS**^.^**^+^ having two reaction phases and melatonin requiring an observation time of 12 min [45]. The results of the evaluation of the antioxidant capacity of wines after treatment with melatonin also showed that due to the synergistic effect between melatonin and classical antioxidants, the highest increase was obtained in FN wine, which could have been due to the strength of the combination between the antioxidant activity of melatonin and the enhancement of antioxidant enzyme activity, but also the content of antioxidant compounds.

## 4. Conclusions

For the first time, melatonin treatment was used in the winemaking of FN and CS red wines, which allowed polyphenolic compounds to be obtained via phenylpropanoid metabolism, and an increase in the concentrations of antioxidant compounds was found to be directly proportional to the concentration of melatonin used. Furthermore, melatonin was found to stimulate the activity of the enzymes PAL and C4H and to significantly alter the expression of specific genes for anthocyanin biosynthesis, in particular, UDP-D-glucose flavonoid 3-*O*-glycosyltransferase. It can be concluded that significantly higher concentrations of resveratrol (50%), quercetin (36%), or cyanidin-3-glucoside (77%) in red wines can be achieved by applying melatonin in the preliminary stage of wine production. It can also be used to produce red wines with increased antioxidant activity (almost 14%). However, further studies are needed to observe the evolution of the profile of polyphenolic compounds or antioxidant activity during the ageing process of the red wines (over at least 12 months) and to determine the appropriate concentration of melatonin required for the treatment of the red wines in order to obtain the expected results.

## Figures and Tables

**Table 1:** Some performance characteristics of the HPLC-MS method for phenolic acid, flavanol, flavonol, and stilbene analysis.

Compound	[M-H]^−^	tR (min.)	The Linear Regression Equations	R	Linearity Range of Response (µg mL^−1^)	LoD (µg mL^−1^)	LoQ (µg mL^−1^)
Gallic acid	169	4.79	A = 48686.49XC − 4680.433	0.9993	0.5–50	0.16	0.30
Caffeic acid	179	15.54	A = 12421.04XC − 879.4463	0.9990	0.5–50	0.19	0.47
Chlorogenic acid	353	14.02	A = 15009.58XC − 434.9512	0.9999	0.5–50	0.13	0.36
Myricetin	317	28.99	A = 58850.16XC − 16277.28	0.9993	0.5–50	0.33	0.45
Quercetin	301	35.89	A = 72301.05XC − 10684.39	0.9997	0.5–50	0.19	0.29
Quercitrin	447	26.89	A = 56594.18XC − 3157.114	0.9998	0.5–50	0.11	0.23
Quercetin 3-β-D-glucoside	463	24.71	A = 47325.40XC − 8096.301	0.9992	0.5–50	0.23	0.38
Rutin	609	23.66	A = 35613.21XC − 747.2902	0.9995	0.5–50	0.11	0.30
(−)-Catechin	289	2.70	A = 190597.3XC − 4539.659	0.9995	0.5–50	0.10	0.29
(+)-Catechin	289	14.23	A = 19405.45XC − 6469.358	0.9999	0.5–50	0.35	0.38
(−)-Epicatechin	289	18.07	A = 13661.42XC − 5979.017	0.9997	0.5–50	0.45	0.47
p-Coumaric acid	163	23.37	A = 232000.5XC − 16316.34	0.9994	0.5–50	0.13	0.29
Naringenin	271	38.91	A = 1086190XC − 360643.9	0.9997	0.5–50	0.35	0.38
Ellagic acid	301	24.10	A = 120801.7XC − 22281.19	0.9993	0.5–50	0.21	0.27
trans-Cinnamic acid	147	2.28	A = 127642.1XC − 6798.598	0.9997	0.5–50	0.17	0.44
Resveratrol	227	32.01	A = 30713.35XC − 5424.285	0.9994	0.5–50	0.23	0.34

**Table 2:** Some performance characteristics of the HPLC-MS method for anthocyanidin and anthocyanin analysis.

Compound	t_R_ (min.)	[M+H]^+^	The Linear Regression Equations	R	Linearity Range of Response (µg mL^−1^)	LoD (µg mL^−1^)	LoQ (µg mL^−1^)
Delphinidin	29.05	303	A = 39532.71XC − 15380.05	0.9995	0.5–50	0.40	0.41
Delphinidin-3-glucoside	24.33	465	A = 2576.836XC − 1070.165	0.9997	0.5–50	0.44	0.49
Peonidin-3-glucoside	28.27	463	A = 41469.73XC − 14707.33	0.9996	0.5–50	0.36	0.38
Cyanidin-3-glucoside	25.87	449	A = 10929.26XC − 4870.150	0.9997	0.5–50	0.46	0.49
Malvidin	34.87	331	A = 71847.46XC − 11253.31	0.9995	0.5–50	0.20	0.30
Malvidin-3-glucoside	28.86	493	A = 5858.430XC − 2110.814	0.9996	0.5–50	0.39	0.45
Petunidin-3-glucoside	26.89	479	A = 279062.4XC − 98044.39	0.9998	0.5–50	0.36	0.39

**Table 3:** The phenolic acid, flavanol, flavonol, and stilbene values obtained by the HPLC-MS method from red wines after 3 months of ageing.

Compound	Control CS Wine Concentration ± SD µg mL^−1^	Treated CS Wine Concentration ± SD µg mL^−1^	Control FN Wine Concentration ± SD µg mL^−1^	Treated FN Wine Concentration ± SD µg mL^−1^
Gallic acid	12.14 ± 0.23	9.13 ± 0.19	16.32 ± 0.28	15.62 ± 0.35
Caffeic acid	4.63 ± 0.19	4.73 ± 0.10	9.79 ± 0.03	10.98 ± 0.04
Chlorogenic acid	11.11 ± 0.53	11.12 ± 0.09	6.25 ± 0.15	7.75 ± 0.25
Myricetin	26.75 ± 0.42	30.20 ± 1.21	25.47 ± 0.55	32.26 ± 0.11
Quercetin	28.91 ± 0.04	30.41 ± 0.14	16.59 ± 0.13	23.07 ± 0.01
Quercitrin	2.09 ± 0.02	2.44 ± 0.20	0.14 ± 0.02	0.16 ± 0.01
Quercetin 3-β-D-glucoside	35.67 ± 0.67	37.64 ± 0.96	3.78 ± 0.24	5.14 ± 0.01
Rutin	2.86 ± 0.04	3.26 ± 0.14	1.30 ± 0.05	1.36 ± 0.02
(−)-Catechin	12.84 ± 0.18	16.38 ± 0.30	15.37 ± 0.13	12.14 ± 0.20
(+)-Catechin	62.01 ± 0.74	75.69 ± 0.87	31.18 ± 0.30	31.92 ± 0.07
(−)-Epicatechin	39.95 ± 0.85	47.80 ± 0.57	40.27 ± 0.86	43.14 ± 0.05
Ellagic acid	8.53 ± 0.13	9.33 ± 0.50	14.40 ± 0.42	16.38 ± 0.38
Resveratrol	9.30 ± 0.27	13.21 ± 0.46	12.85 ± 0.15	19.29 ± 0.35
p-Coumaric acid	10.10 ± 0.22	12.70 ± 0.35	2.10 ± 0.07	3.53 ± 0.14
Naringenin	12.50 ± 0.35	16.60 ± 0.51	10.10 ± 0.13	16.80 ± 0.47
trans-Cinnamic acid	9.13 ± 0.19	12.66 ± 0.29	4.61 ± 0.08	6.72 ± 0.09

SD—standard deviation.

**Table 4:** The anthocyanidin and anthocyanin values obtained by the HPLC-MS method from red wines after 3 months of ageing.

Compound	Control CS Wine Concentration ± SD µg mL^−1^	Treated CS Wine Concentration ± SD µg mL^−1^	Control FN Wine Concentration ± SD µg mL^−1^	Treated FN Wine Concentration ± SD µg mL^−1^
Delphinidin	1.30 ± 0.02	1.34 ± 0.02	1.27 ± 0.03	1.30 ± 0.01
Delphinidin-3-glucoside	804.27 ± 2.6	950.80 ± 1.32	564.31 ± 2.03	688.37 ± 1.7
Peonidin-3-glucoside	25.41 ± 0.28	29.10 ± 0.16	25.23 ± 0.25	31.24 ± 0.37
Cyanidin-3-glucoside	5.21 ± 0.05	7.30 ± 0.06	1.80 ± 0.23	3.19 ± 0.01
Malvidin	23.93 ± 0.6	22.71 ± 0.25	4.27 ± 0.16	4.38 ± 0.13
Malvidin-3-glucoside	668.01 ± 2.19	688.32 ± 2.42	708.74 ± 2.79	708.34 ± 0.97
Petunidin-3-glucoside	93.91 ± 0.31	106.97 ± 0.36	92.75 ± 0.73	104.66 ± 0.34

SD—standard deviation.

## Data Availability

The authors confirm that the data supporting the findings of this study are available within the article.
